# On ‘Organized Crime’ in the illicit antiquities trade: moving beyond the definitional debate

**DOI:** 10.1007/s12117-012-9182-0

**Published:** 2013-01-16

**Authors:** Jessica Dietzler

**Affiliations:** Scottish Centre for Crime and Justice Research, University of Glasgow, College of Social and Political Sciences, Ivy Lodge, 63 Gibson Street, Glasgow, G12 8LR UK

**Keywords:** Organized crime, Transnational crime, Criminal markets, Illicit antiquities, Antiquities trafficking, Routine activity theory

## Abstract

The extent to which ‘organized crime’ is involved in illicit antiquities trafficking is unknown and frequently debated. This paper explores the significance and scale of the illicit antiquities trade as a unique transnational criminal phenomenon that is often said to be perpetrated by and exhibit traits of so-called ‘organized crime.’ The definitional debate behind the term ‘organized crime’ is considered as a potential problem impeding our understanding of its existence or extent in illicit antiquities trafficking, and a basic progression-based model is then suggested as a new tool to move beyond the definitional debate for future research that may help to elucidate the actors, processes and criminal dynamics taking place within the illicit antiquities trade from source to market. The paper concludes that researchers should focus not on the question of whether organized criminals- particularly in a traditionally conceived, mafia-type stereotypical sense- are involved in the illicit antiquities trade, but instead on the structure and progression of antiquities trafficking itself that embody both organized and criminal dynamics.

## Introduction

The last several years of scholarship on the illicit antiquities trade have brought to light a great deal of information about the actors, processes, and realities of the transnational exploitation of illicitly acquired archaeological antiquities. The work of researchers has helped to create a framework for analyzing the exploitation of illicitly acquired archaeological antiquities and the criminal activities involved therein; however, significant gaps in our understanding of the trade, process, and actors remain. Studying the criminal dynamics of the illicit antiquities trade is difficult due to a paucity of available official data and the often filtered, sensationalized, non-specialist media sources that are available; as a result, the extent to which ‘organized crime’ is involved in the illicit antiquities trade is unclear (Mackenzie 2009:52), sometimes debated, but more often assumed. However, the most reliable analyses suggest that organized crime in the illicit antiquities market generally does not appear to be stereotypical Mafia, but in the few cases that it is, it is the exception rather than the rule (Proulx 2010; Nistri 2009:98). Scholars agree that there is a spectrum of ‘organized’ crime, but despite increasing sophistication in the treatment of the term in the literature, the question of whether organized crime (as traditionally conceived) is involved in the illicit antiquities trade still remains. There is limited evidence to support that stereotypical mafia-type organized crime is involved (Mackenzie 2009:48); however, ‘organized crime’ as flexible, variable, opportunistic networks does appear to be involved.

This paper begins with a consideration of looted antiquities generally and the difficulties in estimating the international extent of the problem. Next, the paper considers why ‘organized crime’ terminology matters in the context of looted antiquities, why the terminology is problematic, and how media-perpetuated stereotypes of organized crime have a distracting influence on understanding the nature and extent of organized crime involvement in the trade. A new four-stage progression model is then suggested as a fundamental platform from which to build future research into the precise role of organized crime within the illicit antiquities trade at local, national, and international levels.^1^ In light of this model, the paper concludes that researchers should focus not on the question of whether organized criminals- particularly in a traditionally conceived, mafia-type stereotypical sense- are involved in the illicit antiquities trade, but instead on the structure and progression of antiquities trafficking itself that embody both organized and criminal dynamics.

### Significance and scale of the problem

The scope and significance of the illicit antiquities trade is global, underreported, publicly misunderstood, and has become a crisis of epic proportions with regard to the quality and quantity of our knowledge of human history (Bowman 2008:228–230; Brodie and Renfrew 2005; Brodie and Tubb 2002; Brodie 2011:409; Brodie et al. 2006; Gill and Chippendale 1993; Mackenzie 2009:52; Proulx 2010; Renfrew 2000). The illicit antiquities trade is a highly sophisticated and lucrative business for profit/status-driven individuals, whether collectors, professional dealers, or other middlemen. The trade is not lucrative, however, for the locals or indigenous peoples in source countries ravaged by conflict, political instability, or economic hardship (Bowman 2008:232; Brodie 2002; Hardy 2011:44; 203–204; Mackenzie 2009:52; Sandage 2009). In fact, analysts such as Brodie (1998:1) have calculated that ‘looters’ (local people comissioned by dealers or other middlemen) in source countries receive only around 1 % of the overall profit that is made at the close of sale; Brodie found that over 98 % of profits went to ‘middlemen’ rather than to local diggers in source countries. Source countries are understood to be the starting point of the illicit antiquities trade process, the locations from where archaeological materials are illegally and unsystematically excavated. They are countries known to be rich in archaeological material, whose archaeological sites fall victim to the looting process (unsystematic clandestine excavation) usually as a result of one or all of the following: weak and/or corrupt political infrastructure, military conflict, unstable economic conditions, and lax law enforcement policies and/or funding to curb the problem.

The problem of looted or stolen antiquities is most damaging in politically conflicted and economically depressed regions (Brodie 2011:408–411; Dietzler 2007; Hardy 2011; Manacorda 2009) but is not isolated to conflict regions alone; in fact, there are a number of politically and economically stable countries that also experience theft of archaeological materials for profit; notably England, France, Italy, Germany, and Poland (Sandage 2009). Countries easily fall victim to looting during times of political unrest; as a result of the military conflict and political instability in Afghanistan, for example, there is now a prolific trade in illegally acquired archaeological materials and other antiquities — the exploitation and sale of which constitutes an economic strategy for survival among local people (Schetter 2002:9–10) as well as being reported as a mechanism of ‘terrorist funding’. In such source countries, looting can occur at both publicly known and unknown archaeological sites (Proulx 2010:2). Bribery, falsification of legal documents and other pedigree paperwork (ownership history, etc.), weak law enforcement and/or weak political infrastructure, and other corrupt practices are also typical throughout the entirety of the trafficking process from source to market end (Mackenzie 2009; Sandage 2009). In source, transit, and market countries, official corruption is often both accommodating and frequent, especially when the items are not portable and necessitate the use of heavy machinery (for digging and/or removal), forged export documents, or freight shipping methods (Alder and Polk 2005:100–103; Mackenzie 2009:50).^2^

### Looting & ‘grey’ legitimacy

A high number of antiquities that end up on the market are suspected to have been clandestinely removed from archaeological sites, hidden, and illegally transported (smuggled) across international borders (Proulx 2010; Mackenzie 2005). Most recently-surfaced antiquities on the market have either 1) no find-spot documentation or ownership history, or 2) falsified or vague and unverifiable ownership history, thereby disguising the illicit item under a cloak of licitness and allowing entry into the open market (Alder and Polk 2005; Brodie 2002, 2009a, b, 2011:409). To further complicate matters, the sale of *licitly*-obtained antiquities is legal (Alder and Polk 2002; Alder et al. 2009:126), giving the impression that the trade is altogether a completely licit one. However, considering the illicit means by which most archaeological antiquities are sourced and end up on the market, the seemingly ‘licit’ nature of the trade is confusing, if not deceptive. In fact, anyone who seriously considers the archaeological antiquities market a legitimately and wholly licit one is either uninformed about the realities of the trade or they are willfully ignoring the realities of the trade altogether; usually it is the latter (Brodie 2002; Mackenzie 2009).

The confusing mix of both licit and illicit items on the market in conjunction with the constantly shifting legal status of those items from supply/source country to demand/destination location are two of the main reasons why scholars refer to the market as a ‘grey’ one (Polk 2000; Bowman 2008; Brodie 2011:409; Mackenzie 2009). Though the illicit antiquities trade shares many similarities with other transnational crimes, it is unlike some transnational crimes precisely because of its ‘grey’ nature (Polk 2000; Bowman 2008; Mackenzie 2009). Therefore, due to the illicit means by which the majority of archaeological antiquities on the market are thought to have been acquired, the constantly shifting legal status of the items, in addition to the questionable or total lack of find-spot documentation and/or ownership history, the readiness of the market to embrace illicit antiquities for sale is striking although not entirely surprising considering the “culture of ignorance” (Mackenzie 2009:47) embraced by most dealer communities.

### Scale: official estimates

Despite the illegality of trafficking and selling illicitly acquired archaeological materials, the illicit antiquities trade remains a lucrative and thriving business. However, assessing overall profitability and criminal dynamics of the trade is difficult due to problems with official data (Proulx 2010:2),^3^ in addition to the difficulty in quantifying numbers concerning black market goods by virtue of the illegal, secretive, ‘underground’ nature of trafficking activities. Illicit antiquities crimes often go underreported (if they are reported at all) and, though official data does exist, there is an overall paucity of that data for several reasons (Proulx 2010:1–2). First, national US criminal statistics regarding illicit antiquities crime are problematic because illicit antiquities crimes are often lumped together with and labeled as ‘art crime,’ a phenomenon that bears little resemblance to the acquisition, smuggling, and sale of illicit archaeological materials (Proulx 2010:2).^4^ Secondly, national criminal statistics regarding illicit antiquities may not be entirely representative of the trade because the type of crime recorded is a category which tends to refer to the generic modus operandi of the criminal act as opposed to the “type of object [that is] stolen” (Proulx 2010:2). Moreover, in a 2011 paper, Brodie highlights the gravity of the problem with illicit antiquities data in the statement: “There are *no reliable statistics* describing either the material volume or monetary value of the trade” (p.411) (my emphasis in italics).

Despite all of the problems with official data, there are some figures that do exist and do promote the lucrative nature of illicit antiquities trafficking as an international multi-billion dollar industry (Proulx 2010), although researchers need to be wary of uncritically accepting these estimates which, as with many other transnational criminal markets, tend to be re-circulated to the point of becoming widely known without their sources being robustly appraised. With that caveat in mind, we can note that as commodities, illicit antiquities profits *allegedly* exceed those of other transnational crimes such as narcotics and arms, in terms of value gained from source to market (UNESCO 2011); and according to the United Nations Educational Scientific and Cultural Organization, illicit antiquities are considered to be one of ‘the most persistent illegal trades in the world’ alongside arms, narcotics, counterfeiting, and cybercrime (UNESCO 2011:3). From source to market, the value of illicit antiquities is said to increase as much as “100 fold, a greater growth than that of drugs” (UNESCO 2011:4), possibly due to the fact that archaeological antiquities are a limited resource and “scarce commodity” (Mackenzie 2009:50) and are usually purchased by wealthy high-status individuals or institutions (Chappell and Polk 2009:6; Mackenzie 2009:51), while narcotics and arms can be manufactured at will and purchased by a larger swath of the population. Whatever we make of what must be suspected to be over-hyped estimates of the scale of the problem, or if any of these figures are based in fact, then clearly the market for illicit antiquities is not “shrinking” as some dealers purport (Mackenzie 2009:53); rather, the illicit antiquities market continues to thrive, is highly profitable and fed by illegally acquired archaeological materials which represent an international commodity for which there is a strong existing demand.

### Transnational crimes

The illicit antiquities trade is a dynamic transnational phenomenon (Alder and Polk 2005), at once both global and local — sometimes referred to as ‘glocal’ (Proulx 2010) having grown in complexity and cultural significance over the last several decades. Transnational crimes, in general, have become more complex due to the processes and effects of globalization and the illicit antiquities trade is no exception to the rule (Bowman 2008:225). Globalization brings change (social, political, technological, economic, et al.) and international markets (both legal and illegal) react and adapt to those changes. The market for illicit antiquities is not immune to the effects of globalization and has — like other markets — had to adapt and react. The ease of international travel and information sharing via the internet and other technological means have made geographic considerations somewhat of a non-issue, and criminals are able to exert their influence over increasingly wider areas (Bowman 2008:225–226; Finckenauer 2005:79–80; Paoli 2002:51,69,70; Robinson 2000). In recent years, several scholars have suggested that the trade in illicit antiquities closely resembles — and can be tied to — other illegal activities such as drug smuggling, human trafficking, arms sales, money laundering, terrorism, and ‘organized crime’ (Bogdanos 2011; Bowman 2008:230–231; Brodie et al. 2000; Hardy 2011:204–209; Proulx 2010; Renfrew 2000).

### Organized crime and mafia-colored perceptions

Over the last several years scholars in the ‘small field’ (Proulx 2010:1) of illicit antiquities crime have attempted to ascertain the extent to which ‘organized crime’ is involved in the illicit antiquities trade (Alder and Polk 2002, 2005; Chappell and Polk 2011; Proulx 2010; Mackenzie 2005, 2009, 2011; Tijhuis 2006) but have been challenged by certain factors, particularly the definition of ‘organized crime.’^5^ For example, the field of criminology sees a significant difference between the terms and concepts “Organized Crime” and “organized crime” (Hagan 2006:134). For Hagan, “Organized Crime” refers to hierarchically organized groups, whereas “organized crime” refers to criminal activities that exhibit some degree of organization (ibid.) Understanding whether organized crime (of either type) is involved in illicit antiquities trafficking is important because, depending on which term is used and by whom, it could significantly affect legislative decisions, policy design, and law enforcement responses to the problem. In 2008, as a result of increased scholarly interest, the United Nations (UN) hosted a three-day conference in Italy devoted entirely to the topic: *Organized Crime in Art and Antiquities*.^6^ The consensus of the 2008 UN conference was, in essence, threefold: first, that the involvement of organized crime in illicit antiquities trafficking depends entirely upon what is meant by ‘organized crime;’ second, that it does not appear to be organized in the stereotypical Mafia sense of the term; finally, that there was not enough information at the time to determine the precise role of organized crime in the trade.

The conference conclusions underscore the significant disagreement among scholars as to whether ‘organized crime’ is involved in the trade at all. The disagreement is likely a result of the confusion stemming from the definitional debate behind the meaning of ‘organized crime’ as a concept and frequently misused term, exacerbated by an overall paucity of official data available for study (Brodie 2009a, b:72–73; Proulx 2010). For instance, with regard to the definitional debate on organized crime in the illicit antiquities trade, Proulx (2010) studied the opinions of field archaeologists worldwide in order to elicit their personal experiences, regarding the involvement of organized crime in the antiquities trade, and whether they personally witnessed looting or organized criminals in action in the field. Proulx found that archaeologists’ perceptions and opinions of organized crime were colored by notions of American-Italian mafia, such as the ones represented and sensationalized in popular media (blockbuster movies such as *The Godfather* and television programs such as *The Sopranos*, news stories about La Cosa Nostra, et al.).

Proulx’s findings are consistent with what other criminologists have discussed with regards to the apparent mafia-centered pollution of the ‘organized crime’ concept (Finckenauer 2005:63; Paoli 2002:52). In fact, one of the primary obstacles to understanding the meaning of ‘organized crime,’ is the too-frequent use of the term in reference to criminal activities of transnational scope and presumed association with mafia-centered involvement (Paoli 2002:51). Finckenauer (2005:63) points out that while the most common conception of ‘organized crime’ is synonymous with mafia, ‘organized crime’ and mafia are two different things entirely (p.75). For Finckenauer (p.73), mafia is a socially constructed idea, a “cultural artifact,” that is but one form of so-called ‘organized crime’ (p.74). Proulx’s findings demonstrate a significant problem that researchers studying organized criminal involvement in the illicit antiquities trade face. That is, preconceived notions stemming from media influence can significantly color data and, if used in vain, can be very dangerous. For Paoli (2002:55), the term ‘organized crime’ is “ethnically loaded” and therefore dangerous as it conjures up ideas and images of stereotypical mafia; this could be damaging to certain ethnic communities in terms of their relation to their respective local social networks and governments. Finckenauer (2005:77–78) echoes Paoli’s sentiment, noting the danger in labeling crimes that are organized as ‘organized crime.’

Scholars disagree with each other as to the precise nature of ‘organized crime,’ but are in agreement that there is instead a spectrum of what constitutes ‘organized,’ and that there is not necessarily a single definition that encompasses all manifestations of conduct that is both organized and criminal (Finckenauer 2005:68; Hagan 2006:127; Orlova 2008:99; Paoli 2002:52,60; Von Lampe 2006).^7^ The term ‘organized crime’ is problematic and the concept elusive (Edwards and Levi 2008:364), probably due to the confusing inclusion of the word ‘organized’ (Finckenauer, p.64), and the domination of mafia stereotypes embedded in most people’s perception of the term.^8^ Ideas and definitions of ‘organized crime’ vary widely (Finckenauer 2005:73; Hagan 2006:127) — possibly the reason why many textbooks do not even provide a working definition (Hagan 2006:129–130). For Hagan (p.128), a universal working definition does not yet exist, but there is a need for one as definitions affect law and policy-making. While Hagan is correct that a definition is needed, ‘organized crime’ (whatever it is) does not fit well into a “tight legislated definition” because the phenomenon itself is “amorphous” and “fluid,” thanks to the processes and effects that globalization has on criminal activity (Orlova 2008:134). The term is at best problematic and, at worst, downright harmful in that it could be “used to describe almost any serious criminal occurrence” (ibid.) involving more than one individual. It is for this reason that Orlova (2008:133) suggests taking into account the socio-economic factors (unemployment, poverty, ethnic marginalization, et al.) that may influence or even necessitate organized criminal activity on the part of some community members. This is an especially important point when considering looting activity in source countries ravaged by war or political distress. Since most criminal activities are shaped by local socio-economic and cultural contexts, national legislation should be specifically, albeit very carefully, tailored to fit local situations (p.134).

Given the potential implications of the ‘organized crime’ label, the responsibility that weighs upon the organized crime researcher is heavy, as it is published material that is cited and used by policy-makers when forming or enacting new legislation or by law enforcement designing useful responses to the problem. Unfortunately, still, news stories pour out of the media, books and articles are written, non-existent figures are cited as ‘fact,’ and the proliferation of the polluted, stereotyped, ethnically-loaded mafia-centered concept of ‘organized crime’ persists; the trade in illicitly-obtained antiquities is no exception. In fact, popular media have been especially instrumental in perpetuating this stereotypical mafia-type organized crime concept within the context of antiquities. Manacorda and Chappell (2011) give an example of the questionable use of the term ‘organized crime’ in the field of art crime where they observe one writer presenting un-supporting ‘facts’ about the relationship between art crime, organized crime, and terrorism without an apparent scientific basis to do so (see Manacorda and Chappell 2011:3–5 referring to Charney 2009). Consider, however, how easy it is for ‘cited’ opinion to be taken at face value and, worse yet, even referenced as ‘cited’ fact in subsequent scholarship, policy formation, and even courts of law. Herein lie the very serious implications of the use of the term ‘organized crime.’

### Moving beyond the conflated concept

We can shift our focus away from the definitional debate and instead toward understanding more thoroughly the structure of the illicit antiquities trade as a transnational market that is both organized and criminal by creating a framework of analysis based on Routine Activity Theory (RAT) and supported by Marcus Felson’s (2006) ‘events, sequences, and settings’ approach to understanding organised crime.^9^ RAT is a neo-classical and spatially-oriented methodological approach for analysing criminal activities generally and is useful for the purposes of understanding the structure of illicit antiquities trafficking as a transnational phenomenon. RAT proposes that crime arises through the conjunction of three fundamental components: 1 — a *suitable target*; 2 — a *motivated offender*; and 3 — the *absence of capable guardianship*. In the case of the illicit antiquities market, a *suitable target* can be understood to be antiquities (e.g., the target desired — that is suitable for a variety of reasons. (see Mackenzie 2009)); a *motivated offender* can be any person whose choice to engage in criminal activity is based on rational decision-making and is motivated enough to carry out the offense; the *absence of capable guardianship* is the idea that at any stage in the process of trafficking an offense can be successfully carried out by a motivated offender who has identified a suitable target in an environment that lacks surveillance (bystanders, CCTVs, police, et al.) and makes a rational decision to act.

Building on RAT, Felson (2006) provides another useful approach to understanding and dealing with organized crime, suggesting that a new “intellectual image of criminal cooperation is needed” (p.8) in order to move beyond the definitional debate and arrive at an appropriate understanding and response to the phenomenon. According to Felson (2006:7), organized crime is dependent upon poorly managed societies and necessitates recurrent local social settings where criminals can meet to conduct business or recruit other criminals (p.10). Felson asserts that the organizing principle of organized crime can be found in the ‘events, sequences, and settings’ of criminal activity (p.8) and, if researchers look at the events, sequences, and settings more closely, our intellectual understanding of organized crime can be greatly enhanced (ibid.). Therefore, it is important to focus on events, the settings they occur within, and their sequences; in this way, the structure and continuity of criminal activities, whether loosely or tightly organized, can be studied by looking at the settings where offenders converge (p.10). Once identified, these ‘convergence settings’ offer unique opportunities for understanding “the structure of criminal cooperation, even when [individual actor] participation is unstable” (Felson, p.9). Offender participation is not stable because actors involved in criminal processes are not necessarily static fixtures; relationships between contacts (at any point in the process) sometimes break down due to arrests, lack of trust, et al. (p.13). It is for this reason that Felson suggests focusing on the settings where actors converge, as opposed to focusing on the actors themselves, if one wishes to understand the nature of organized crime (p.10).

### Four-stage sequence of progression

RAT, when combined with Felson’s (2006) ‘events, sequences, and settings’ approach to understanding organized crime, provides an intellectual framework that can be used as an effective tool of analysis for the illicit antiquities trade as an organised global phenomenon. By adopting this methodological approach and considering Mackenzie’s (2009, 2011) ‘market as both organized and criminal’ perspective, it may thus be helpful to construct a simple progressive framework from which to build further research into the connections between illicit antiquities trafficking and organized crime by focusing not on the actors themselves but instead on the progression of the trade and its basic sequences from source to market. The benefit of a general progression-based model is that it provides a broad chartable view of the entire process of illicit antiquities trafficking, while simultaneously allowing for specific stages of the process to be studied as separate ‘ecosystems’ (Felson 2006) in order to ascertain the extent of organization and criminal involvement. It thus encourages scholarly focus to fall first and foremost on the concept of a market that is both criminal and organized in its structure, functioning, and progression, and then on the actors themselves who may or may not be ‘organized criminals’ in even a fluid, elastic sense. The model allows actors to ‘fit’ into general sequences of the progression, whether or not those actors change or remain the same. Moreover, such a model is useful for microscopic analysis of criminal connections and activities at different stages throughout the trade. Overall the progression model, focused on the criminal elements and organizational structure and sequence of antiquities trafficking, rather than on the question of whether actors involved embody ‘organized crime’ which has preoccupied much scholarship as discussed above, embraces a more interdisciplinary, nuanced approach to understanding the organized criminal elements of the illicit antiquities trade. The four-stage progression model in illicit antiquities trafficking can thus be charted as follows in Table 1 below.

**Table 1:**
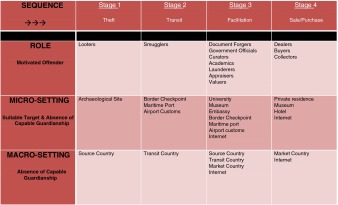
Four-stage progression model

The four-stage progression model provides a fundamental framework for understanding the basic sequences of progression that occur within the illicit antiquities market from source to demand end. Its importance is in this provision of a framework, and the categories are filled in the model presented here for illustrative purposes only rather than attempting an exhaustive exposition of the roles and settings in the market, which is not the aim of this paper. As noted above, it also shifts the research emphasis from consideration of individuals as both organized and criminal (‘organized criminals) to a focus on the structure and processes of trafficking as both organized and criminal (‘organized crime’). The model is thus a simple framework with at least three potential implications. First, the model helps to articulate the processes, events, settings, and actors involved in each of the four stages. Second, the data retrieved from analyzing each of the four stages *separately* could elucidate the level and extent of organized criminal dynamics taking place within each stage, filling in the knowledge gaps on how such cooperation takes place from one stage to the next. Third, the data collected for each of the four stages could help local, national, and international law enforcement design more effective and locally-tailored responses to the problem respecting each stage’s unique social considerations and environment. Clearly, the model allows for more focus on the market itself as both organized and criminal, which is independent of whether the individuals involved themselves could be considered both organized and criminal (Proulx 2010; Mackenzie 2009). Situated within this progression model, the types of illegal activities taking place within the illicit antiquities trade can thus be considered ‘organized crime’ by virtue of their complexity and the ingenuity demonstrated therein (Chappell and Polk 2009:2); in fact, the illicit antiquities market is “…*in itself* an example of organized crime” (Mackenzie 2009:41;59), by virtue of the organized structure of relationships between actors and criminal activities (Paoli 2002:69). Therefore, if the market is itself both criminal and organized, then the individuals participating in each stage of the progression from source to market are also criminal and organized to some degree (however loosely or tightly structured) (Paoli 2002:61;67–68). Moreover, if ‘organized crime’ in the stereotypical mafia sense is involved in illicit antiquities trafficking, it may not matter much in terms of the supply and demand of the objects themselves (Mackenzie 2009); that is, organized crime involvement is not going to change the demand for illicit antiquities in market countries, therefore — as with any other criminal market — the supply will be driven as long as the demand persists (Paoli 2002:88).^10^

In light of the progression-based model described above, and provided that antiquities trafficking is understood as a market that is in itself both organized and criminal, the 2003 UN Convention on Transnational Organized Crime (UN 2004) arguably offers some of the most significant enforcement tools to combat the trafficking of illicit antiquities. The convention tried to standardize definitions of organized crime, criminalization of activities, and guidelines for international cooperation and legal assistance. Illicit antiquities dealers could thus be understood, by virtue of their association with other criminals trafficking illicit antiquities through the progressive pipeline, as complicit members of this organized criminal market, regardless of whether they operate in a “culture of ignorance” or not. Dealers, by virtue of their willingness to participate in the selling of illicit antiquities, take advantage of open borders (through their willingness to cooperate and purchase items from actors who are physically taking advantage of open borders), and the supply of their commodities rely explicitly upon the weaknesses of political and economic infrastructure in source countries. If the economic exploitation of looters and archaeological sites in source countries can be understood as ‘injustice’ (UN Millennium Declaration 2000), then dealers, by virtue of their complicitness and power in driving the market, can therefore be understood to be unwitting oppressors helping to create that injustice, regardless of their physical or cultural distance from looters or archaeological sites in source countries.

## Conclusion

The words we use to discuss social problems like looting, and the meanings and ideologies we ascribe to those words (like ‘organized crime’), shape attitudes and perceptions which drive policy and law. As Mackenzie (2005:1) argues, “before we can talk of how best to regulate the market, we must be sure of the existence and nature of the problem we wish to address.” For Chappell and Polk (2009:10), thinking of illicit antiquities trafficking as ‘organized crime’ is important because it emphasizes the complexity of the trade overall, as well as the challenges in designing appropriate responses to the problem. Thinking about illicit antiquities within the realm of organized crime changes how we frame the problem and respond to it, but flexibility is necessary with what is meant by ‘organized crime.’ The exploitation of local impoverished looters in source countries, in conjunction with the exploitation of the world’s archaeological landscape for personal profit, is an affront to human dignity and human history. In their search for tantalizing ancient artifacts, dealers and collectors are now faced with an interesting dilemma; that is, in their quest to admire and collect rare pieces of human history they are at the same time unwittingly — and sometimes knowingly — destroying it through criminal and divisive means at an unconscionable and alarming rate.

Though definitional problems and debate still surround the meaning of so-called ‘organized crime’ and its involvement in illicit antiquities trafficking, research must move beyond the definitional debate, and must understand and accept that the illicit antiquities market is both organized *and* criminal in order for new law enforcement responses or policy-making to have any lasting effect on the trade. Approaching and studying the illicit antiquities trade through an ‘events, sequences, and settings’ approach (as suggested in the four-stage progression model) allows for the basic sequences of progression to be analyzed microscopically as individual ecosystems and the links between them explicated in order for the enhancement of our understanding of the internal, hidden dynamics of the trade from source to market. The illicit antiquities trade is a transnational market connecting supply and demand settings and events, via transit, trade and facilitation mechanisms. Therefore, in order to stop the devastation of archaeological landscapes, and to stop the economic exploitation of impoverished people in struggling societies hard-hit by the ravages of conflict or political distress, future legislation and policy-making should begin by considering the routine activities of the market in terms of these analytical categories.

## Footnotes

1 The author will henceforth use the term ‘organized crime’ in its ambiguous form throughout this paper in order to further underscore the definitional issues inherent within it.

2 The author met with an individual who hails from a European country and works as a full-time freight shipping specialist at a large European port. According to this source, shipping illicit antiquities from one country’s port to another is “relatively easy,” as “freight shipping is the preferred method for these kinds of [illicit antiquities] things, if this is what you are trying to do” (Personal communication, anonymous European freight shipping specialist, 22 May, 2011).

3
*Official data* i.e., military records, law enforcement reports, national criminal statistics, etc.

4 ‘Art crime’ generally refers to the theft of known, identifiable art works from established museums, institutions, or private collections (Tijhuis 2006:129; Proulx 2010:2).

5 Proulx (2010:1;5–6) rightly points out that there is but only a small handful of scholars who *specialize* (e.g., put forth significant criminological contributions) in researching the criminal aspects of the illicit antiquities trade.

6 The International Scientific and Professional Advisory Council of the United Nations Crime Prevention and Criminal Justice Programme (ISPAC) hosted a conference titled *Organized Crime in Art and Antiquities* in Courmayeur Mont Blanc, Italy (12–14 December 2008). The conference was attended by 145 experts from 27 different countries; the proceedings have been published and the information can be found in the References section of this paper.

7 For more information on scholarly disagreement as to the precise nature of ‘organized crime,’ see, for example: Abadinski 1994:6; Albini 1971:37;49; Finckenauer, 2005:81; Ianni 1975:14; Task Force Report: Organized Crime, The President’s Commission on Law Enforcement and Administration of Justice (1967); United Nations, 2000:article 2; 1968 Omnibus Crime Control and Safe Streets Act: p.14, retrieved 21 April 2012 online at: http://transition.fcc.gov/Bureaus/OSEC/library/legislative_histories/1615.pdf

8 Paoli (2002:52) provides an insightful breakdown of how the concept of organized crime became as conflated as it is today, discussing the American debate’s significant influence on the concept (with regards to media sensationalism and mafia, etc.), how the concept was imported into Europe, and its current influence on national and international policy–making and law enforcement responses.

9 For more information about RAT, see for example: Clarke and Felson (1993); Felson (2002).

10 It is for this reason that Mackenzie (2009:55–58) suggests that future policy-making should focus on implementing demand-reduction strategies; presently, however, effective public policy responses are significantly lacking (Chappell and Polk 2009:7). In fact, regulatory controls are low across the entire spectrum of the trade; archaeological site security is relatively non-existent and, more importantly, most countries do not require antiquities dealers to be licensed (Mackenzie 2009:50–51).
